# Evaluation of ABCA1 gene polymorphism as a prognostic index of fibrosis progression in NAFLD patients

**DOI:** 10.1002/edm2.394

**Published:** 2022-11-29

**Authors:** Samar E. Ghanem, Maha M. Elsabaawy, Mervat M. Abdelkareem, Marwa L. Helal, Warda Othman, Mahitab Elsayed, Dalia M. Elsabaawy, Ashraf El Fert

**Affiliations:** ^1^ Department of Clinical Biochemistry and Molecular Diagnostics National Liver Institute, Menoufia University Shebeen El‐Koum Egypt; ^2^ Depatment of Hepatology and Gastroenterology National Liver Institute, Menoufia University Shebeen El‐Koum Egypt; ^3^ Department of Clinical pharmacy, Faculty of Pharmacy Modern technology for technology and information university Shebeen El‐Koum Egypt; ^4^ Department of Clinical pharmacy, Faculty of Pharmacy Menoufia University Shebeen El‐Koum Egypt

**Keywords:** ABCA1, fibrosis, nonalcoholic fatty liver disease, single nucleotide polymorphism

## Abstract

**Introduction:**

It had been evident that non‐alcoholic fatty liver disease (NAFLD) is the new era epidemic. Despite emergence of many drugs on the pipeline that considered candidates to cure NAFLD/NASH, the critical need for defining the cohort liable to fibrosis progression is yet unmet.

**Aim:**

Evaluate ABCA1 (rs1800977) genotyping as a noninvasive predictor of liver fibrosis severity.

**Materials and Methods:**

This study included 118 liver biopsy‐proven NAFLD‐patients. According to Metavir‐fibrosis‐staging, cases were divided to early fibrosis (74 cases), and 44 cases with significant fibrosis (>F2), added to 49 healthy control subjects. All patients were subjected to liver function tests, lipids profile, triglyceride TG index, and hepatic steatosis index (HSI) and real‐time PCR ABCA1 SNP (rs1800977).

**Results:**

Significant differences in transaminases (*p* > .05), albumin (*p* < .009), cholesterol (p0.03), low density lipoproteins (LDL) (0.006), triglycerides (*p* < .001), HSI (*p* < .001), FIB4 (*p* < .001) and APRI (*p* < .001) were reported in those with significant than early fibrosis and control groups. CC was the most prevalent in significant (36.4%) than early fibrosis (13.5%) and control groups (8.2%), with prevalence of C allele in significant fibrosis (*p* ≤ .003). Univariate analysis revealed that DM (*p* ≤ .001), TG index (*p* ≤ .022), cholesterol (*p* ≤ .03), HSI (*p* ≤ .006), LDL (*p* ≤ .02), HDL (*p* ≤ .01), APRI (*p* ≤ .02) and CC genotype (*p* ≤ .005) were the main factors affecting fibrosis progression in NAFLD. However multivariate analysis proved only the role of HSI (*p* ≤ .005), LDL (*p* ≤ .02), HDL (*p* ≤ .003) and CC genotype (*p* ≤ .03) in predicting fibrosis severity.

**Conclusion:**

Dyslipidemias, hepatic steatosis index and ABCA1 (rs1800977) gene polymorphism CC genotype; were the only independent predictors of advanced fibrosis in NAFLD‐patients.

## Graphical abstract

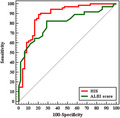

## 1 INTRODUCTION

Nonalcoholic fatty liver disease (NAFLD) prevalence is steeply rising in association with the world rise in type 2 diabetes mellitus and obesity.^1^ The primary stage of NAFLD development is fat accumulation without critical aggravation or hepatocellular injury. In around 10%–25% of subjects, the disease advances to non‐alcoholic steatohepatitis (NASH), described by histological lobular aggravation and hepatocyte expanding.^2^ In about 20% of patients with NASH, the condition advances more leading to liver fibrosis, which might advance to cirrhosis, and its related complications.^3^ The gold standard test for diagnosis, and staging, of NAFLD related fibrosis is liver biopsy.^3^ Different noninvasive markers are available for diagnosis as abdominal ultrasonography, transient elastography, computed tomography, magnetic resonance imaging and Xenon–133 liver scan.^4^ However, there is still unmet need for more accurate non‐invasive predictor of NAFLD‐related liver fibrosis progression.

The ATP binding cassette subfamily A member 1 (ABCA1) gene is located on chromosome 9 (9q31) and contains 58 exons.^5^ ABCA1 is 2261‐amino acids membrane protein that contains 12 transmembrane domains and mediates cholesterol efflux from cell to lipid‐free apolipoprotein A‐I (apoAI) and apolipoprotein E (apoE).^6^ Also, ABCA1 is an important regulator of (HDL) and reverse cholesterol transport.^7^ It is known to be associated with a reduced HDL cholesterol and familial hypoalphalipoproteinemia (FHA), indicating that ABCA1 polymorphism might affect lipids metabolism.^8^ The defined role of ABCA1 in pathogenesis of NAFLD might suggest a confluence between ABCA1 single nucleotide polymorphisms (SNP) and severity of NAFLD along with staging of fibrosis.

## 2 SUBJECT AND METHODS

This study was conducted on 118 consecutive NAFLD patients who were recruited from the Hepatology clinic, National Liver Institute, Menoufia University, from September 2018 to January 2020. Patients were divided according to fibrosis stage by Metavir score into 2 groups: 74 early fibrosis patients (F1, F2) (group I), and 44 with significant fibrosis (F3, F4) (group II). In addition to an apparently healthy 49 subjects with matched age and gender as a control group (group III). The study was approved by the Institution's ethical committee, and a written informed consent was taken from all participants enrolled in the study.

## 3 INCLUSION CRITERIA

Liver biopsy proofed NAFLD patients.

## 4 EXCLUSION CRITERIA

1. Age less than 18 years.
2. Patients with decompensated liver disease.
3. Seropositive for HIV, HCV, HBV or any other liver disease.
4. Significant history of cardiovascular and neuropsychiatric diseases.
5. Patients with hepatocellular carcinoma or any other malignancy.
6. Patients with abnormal thyroid function tests.
7. All cases on drugs lowering lipids were of course excluded.

## 5 ALL PATIENTS AND CONTROL GROUPS WERE SUBJECTED TO THE FOLLOWING

Full history taking, complete clinical examination and laboratory investigations including: ‐.

### 5.1 Liver function tests

Serum transaminases (aspartate transaminase (AST) and alanine transaminase (ALT), gamma glutamyl transpeptidase (ɣ‐GT), alkaline phosphatase (ALP), total bilirubin (TB), albumin (Alb), total proteins (TP) and prothrombin time and concentration (PT and PC%).

### 5.2 Viral markers

Hepatitis B surface antigen (HBsAg), Hepatitis C Antibody (HCV‐Ab).

## 6 ALL FIBROSIS SCORES WERE CALCULATED AS FOLLOWING

**Calculation of APRI** by [(AST/upper limit of normal × 100)/platelet count], where, non‐ significant fibrosis (<F2): <0.7, significant fibrosis (≥F2‐ < F4): 0.7‐.^9^

**Calculation of FIB‐4** by (Age × AST/platelet count × sqr ALT) [12]. Non‐significant fibrosis (<F2) was identified as FIB4 < 1.45, significant fibrosis (≥F2‐ < F4): ‐.^9^

**Hepatic steatosis index (HSI)** [13] HSI = 8 × ALT/AST + BMI + 2, if DM; +2, if female with values <30 ruling out and values >36 ruling in steatosis.^9^

### 6.1 Radiological examination

Abdominal ultrasonography and/ or CT, MRI elastography, and transient elastography (fibroscan).

### 6.2 Molecular analysis

Genomic DNA was extracted from ethylenediaminetetraacetic acid‐anticoagulated (EDTA) whole blood using a spin column method according to the manufacturer protocol (QIAamp Blood Kit, Qiagen).

Genotyping of ABCA1 (rs1800977) was done using the TaqMan allelic discrimination Assay technique which detects variants of a single nucleic acid sequence.

The allelic discrimination assay classifies unknown samples as follows:

1. Homozygote (one allele only was detected CC or TT).
2. Heterozygotes (both alleles were detected CT).

Reaction master mix for amplification was (total volume 20 ul) constituted of.

0.5ul of genotyping assay (primers and probe) 40x purchased from Thermo scientific10 ul of genotyping qPCR Master Mix ((2×), Thermo Fisher Scientific, MA, USA) and 3.5 ul of DNAse‐free water and 6 ul (0.1 μg/μl) of genomic DNA template was added. For the negative control reaction, 6uL of DNAse‐free water was added. Genotyping was performed on real time fast 7500 (Applied Biosystems Thermo Fisher Scientific Inc., Life Technologies TM, CA, USA).

The cycling parameters were set by initial denaturation at 94°C for 4 min, 40 cycles of denaturation at 94°C for 30 s, annealing and extension at 60°C for 1.5 min and a final extension step at 72°C for 3 min.

### 6.3 Statistical analysis

Data were fed to the computer and analysed using IBM SPSS software package version 20.0. (Armonk, NY: IBM Corp). The Kolmogorov–Smirnov was used to verify the normality of distribution of variables; comparisons between groups for categorical variables were assessed using Chi‐square test (Fisher or Monte Carlo). Student *t*‐test was used to compare two groups for normally distributed quantitative variables while ANOVA was used for comparing the four studied groups and followed by Post Hoc test (Turkey) for pairwise comparison. Mann–Whitney test was used to compare between two groups for not normally distributed quantitative variables. Kruskal–Wallis test was used to compare different groups for abnormally distributed quantitative variables and followed by Post Hoc test (Dunn's for multiple comparisons test) for pairwise comparison. Hardy–Weinberg the population of the studied sample was explored to find its equilibrium with Hardy–Weinberg equation. Logistic Regression was used to detect the most independent factor for affecting patient. Odds ratio (OR) was used to calculate the ratio of the odds and 95% Confidence Interval of an event occurring in one risk group to the odds of it occurring in the non‐risk group. Receiver operating characteristic curve (ROC) was used to determine the diagnostic performance of the markers, area more than 50% gives acceptable performance and area about 100% is the best performance for the test. Significance of the obtained results was judged at the 5% level.

## 7 RESULTS

This study shows that all the studied subjects (early fibrosis NAFLD, significant fibrosis and control) were matched in age and gender (*p* > .05) (Table 1).

**TABLE 1:** Comparison between the early, advanced fibrosis, and control groups

	Group I (*n* = 74)	Group II (*n* = 44)	Group III (*n* = 49)	Test of Sig. (*p*)	I vs. II	I vs. III	II vs. III
Sex male	37 (50%)	26 (59.1%)	26 (53.1%)	χ2 = 0.918 (0.632)	>.05	>.05	>.05
Female	37 (50%)	18 (40.9%)	23 (46.9%)				
Age m ± sd.	49.6 ± 11.5	50.9 ± 9	47.5 ± 5.5	F = 1.604 (.204)	>.05	>.05	>.05
ALT m ± sd.	45.9 ± 23.4	50.9 ± 22	25.2 ± 4.9	H = 55.30* (<.001)*	.143	<.001	<.001
AST m ± sd.	39.3 ± 18.2	45.3 ± 31.3	25.7 ± 6.9	H = 23.77* (<.001)*	.641	<.001	<.001
Albumin ± sd.	4.4 ± 0.4	4.3 ± 0.4	4.5 ± 0.3	F = 4.827* (.009)*	.255	.155	.006*
HG m ± sd.	13.3 ± 1.3	12.1 ± 1.8	12.6 ± 1.5	F = 8.253* (<.001)*	<.001*	.048*	.288
Platelets m ± sd.	229.3 ± 45.4	207.6 ± 51.6	302.6 ± 60.3	F = 45.21* (<.001)*	.074	<.001	<.001
Cholesterol m ± sd.	214.5 ± 63.1	237.7 ± 43.9	146.8 ± 124.1	H = 75.82* (<.001)*	.039*	<.001	<.001
TG m ± sd.	177.7 ± 99	178.3 ± 11.5	109.1 ± 18.7	F = 18.16* (<.001)*	.999	<.001	<.001
HIS m ± sd.	44.2 ± 5.8	47.8 ± 7.3	36.7 ± 4.4	F = 44.58* (<0.001)*	.004*	<.001	<.001
LDL m ± sd.	107.5 ± 20.5	132.2 ± 72.68	69.8 ± 24.1	F = 26.71* (<.001)*	.006*	<.001	<.001
HDL m ± sd.	54.8 ± 18.4	71.4 ± 48.3	58.5 ± 10.3	H = 4.334 (.114)	>.05	>.05	>.05
Apri Score m ± sd	0.44 ± 0.21	0.59 ± 0.46	0.22 ± 0.07	H = 57.86* (<.001)*	.551	<.001	<.001
FIB‐4 index m ± sd	1.30 ± 0.52	1.73 ± 1.40	0.83 ± 0.25	H = 33.45* (<.001)*	.673	.001*	<.001

Abbreviations: FE, Fisher Exact; Group I, NAFLD (early fibrosis); Group II, Significant fibrosis; *p*, *p* value for comparing between the two studied groups; *t*, Student *t*‐test; *U*, Mann Whitney test; χ^2^, Chi square test.

*Statistically significant at *p* ≤ .05.

Regarding to the biochemical and clinical characteristics of the enrolled subjects, a highly significant elevation of ALT, AST in group II than I and III (*p* < .001), higher lipids profiles (*p* < .001). While platelets (*p* < .001) and albumin were found to be significantly lower in group II (*p* < .001) (*p* = .006), respectively (Table 1).

In advanced fibrosis, 24 patients (54.5%) were diabetic, while only 8 (18.2%) were hypertensive. Albi and TG scores were increased in advanced fibrosis group (*p* < .006, *p* < .001), respectively. Fibroscan readings and fibrosis indices were higher in group II than group I (*p* < .001) (Table 2).

**TABLE 2:** Comparison between early and advanced fibrosis groups

	Group I (*n* = 74)	Group II (*n* = 44)	Test of Sig.	*p*
BMI (kg/m^2^) m ± sd	33.2 ± 4.9	34.3 ± 2.8	*t* = 1.329	.187
DM	18 (24.3%)	24 (54.5%)	*χ* ^2^ = 10.99*	.001*
HTN	2 (2.7%)	8 (18.2%)	*χ* ^2^ = 8.524*	^FE^ *p* = .005*
FBS m ± sd.	106.6 ± 40.3	114.8 ± 44.2	*U* = 1172.50*	.011*
HbA1c4.59				
m ± sd.	5.7 ± 1.2	6 ± 1.2	*t* = 1.107	.270
TG index				
TG m ± sd.	8.9 ± 0.7	9.2 ± 0.3	*t* = 2.815*	.006*
ALBI score m ± sd.	−0.40 ± 0.07	−0.48 ± 0.07	*U* = 719.0*	<.001*
Fibroscan F0	17 (23%)	0 (0%)		
F1,F2	57 (77%)	27 (61.4%)	*χ* ^2^ = 39.65*	<.001*
F2‐F3, F3, F4	0 (0%)	17 (38.6%)	*χ* ^2^ = 10.363*	.001*

Abbreviations: FE, Fisher Exact; Group I, NAFLD (early fibrosis); Group II, Significant fibrosis; *p*, *p* value for comparing between the two studied groups; *t*, Student *t*‐test; *U*, Mann Whitney test; *χ* ^2^, Chi square test.

*Statistically significant at *p* ≤ .05.

Genotype distribution of ABCA1 SNP (rs 1,800,977) in control group was in accordance with the Hardy–Weinberg equilibrium (*p* = .690) (Table 3). This study shows that 10 patients (13.5%) were CC (wild) genotype carriers in early fibrosis NAFLD, 16 (36.4%) in significant fibrosis and 4 (8.2%) in control (Table 3). CT carriers were 31 (41.9%) in early fibrosis NAFLD, 16 (36.4%) in significant fibrosis and 22 (44.9%) in control and TT (mutant) carriers were 33 (44.6%) in early fibrosis NAFLD, 12 (27.3%) in significant fibrosis and 23 (46.9%) in control subjects (Table 3). There was a significant difference in genotypes of ABCA1 rs1800977 between different groups (*p* = .003) (Table 3). In the current study, the distribution of C allele was 51 (34.5%) in early fibrosis NAFLD, 48(54.5%) in significant fibrosis and 30 (30.6%) in control and the distribution of T allele was 97 (65.5%) in early fibrosis NAFLD, 40(45.5%) in significant fibrosis and 68 (69.4%) in control (Table 3). There was a significant difference in CC genotype distribution between NAFLD patients and late fibrosis (*p* = .005, OR = 4.400), and between late fibrosis and control (*p* = .002* OR = 7667). Regarding allelic distribution, there was difference between early fibrosis and late fibrosis (*p* = .002), between late fibrosis and control (*p* = .001) (Table 3 and Table 4). There was a significant difference in CC genotype distribution of ABCA1 rs1800977 between the NAFLD patients and late fibrosis (*p* = .005, OR = 3.6) and between late fibrosis and control (*p* = .002* OR = 6.429) regarding recessive model (Table 4).

**TABLE 3:** Comparison between the three studied groups according to genotype

	Group I (*n* = 74)	Group II (*n* = 44)	Group III (*n* = 49)	*p*_1_	*p*_2_	*p*_3_
Genotype						
TT	33 (44.6%)	12 (27.3%)	23 (46.9%)	.012*	.657	.003*
TC	31 (41.9%)	16 (36.4%)	22 (44.9%)			
CC	10 (13.5%)	16 (36.4%)	4 (8.2%)			
χ^2^	0.390	3.1289	0.158			
^HW^p	0.532	0.077	0.690			
Allele						
T	97 (65.5%)	40 (45.5%)	68 (69.4%)	.002*	.530	.001*
C	51 (34.5%)	48 (54.5%)	30 (30.6%)			

Abbreviations: Group I, NAFLD (early fibrosis); Group II, Significant fibrosis; Group III, Control; *p*_1_, *p* value for Chi square test for comparing between Group I and Group II; *p*_2_, *p* value for Chi square test for comparing between Group I and Group III; *p*_3_, *p* value for Chi square test for comparing between Group II and Group III; *χ* ^2^(^HW^p), Chi square for goodness of fit for Hardy–Weinberg equilibrium (If *p* < .05 ‐ not consistent with HWE).

*Statistically significant at *p* ≤ .05.

**TABLE 4:** Comparison between the three studied groups according to genotype

	Grp. II vs. Grp. I®			Grp. I vs. Grp. III®			Grp. II vs. Grp. III®		
	II/I	*p*	OR (CI. 95%)	I/III	*p*	OR (CI. 95%)	II/III	*p*	OR (CI. 95%)
Genotype									
TT	12/33		1.000	33/23		1.000	12/23		1.000
TC	16/31	.443	1.419 (0.580–3.473)	31/22	.963	0.982 (0.458–2.106)	16/22	.493	1.394 (0.539–3.603)
CC	16/10	.005*	4.400 (1.571–12.32)	10/4	.394	1.742 (0.486–6.241)	16/4	.002*	7.667 (2.091–28.11)
Dominate									
TT®	12/33		1.000	33/23		1.000	12/23		1.000
TC + CC	32/41	.063	2.146 (0.958–4.807)	41/26	.798	1.099 (0.533–2.268)	32/26	.053	2.359 (0.989–5.624)
Recessive									
TT + TC®	28/64		1.000	64/45		1.000	28/45		1.000
CC	16/10	.005*	3.657 (1.477–9.052)	10/4	.365	1.758 (0.519–5.958)	16/4	.002*	6.429 (1.950–21.19)
Allele									
T®	40/97	.002*	1.000	97/68	.530	1.000	40/68	.001*	1.000
C	48/51		2.282 (1.331–3.914)	51/30		1.192 (0.690–2.060)	48/30		2.720 (1.492–4.959)

*Statistically significant value.

Prediction of factors affecting fibrosis progression in NAFLD, univariate Logistic regression analysis had defined DM, lipids(serum cholesterol, LDL,HDL), TG index,HSI, APRI, FIB4 scores and CC genotype of ABCA1 rs1800977 (*p* < .05). While multivariate logistic regression analysis had nominated HSI, lipids and CC genotype of ABCA1 rs1800977 as independent predictors of fibrosis progression in NAFLD (*p* < .05) (Table 5).

**TABLE 5:** Univariate and multivariate Logistic regression analysis for the parameters affecting significant fibrosis groups

	Univariate		Multivariate^a^	
	*p*	OR (95%C.I)	*p*	OR (95%C.I)
Male	.339	1.444 (0.679–3.071)		
Age (years)	.525	1.012 (0.976–1.048)		
BMI (kg/m^2^)	.190	1.061 (0.971–1.158)		
DM	.001*	3.733 (1.684–8.278)	.160	2.184 (0.734–6.494)
Fasting Bl S	.302	1.005 (0.996–1.014)		
HbA1c4.59	.274	1.171 (0.883–1.553)		
TG index	.022*	2.307 (1.127–4.722)	.401	1.617 (0.527–4.964)
AST	.196	1.010 (0.995–1.026)		
ALT	.254	1.010 (0.993–1.026)		
Albumin	.133	0.457 (0.165–1.270)		
S cholesterol	.037*	1.007 (1.000–1.014)	.138	1.009 (0.997–1.020)
TG	.968	1.000 (0.995–1.005)		
HIS	.006*	1.090 (1.025–1.159)	.009*	1.116 (1.027–1.212)
LDL	.022*	1.012 (1.002–1.023)	.016*	1.017 (1.003–1.031)
HDL	.012*	1.015 (1.003–1.027)	.002*	1.028 (1.010–1.046)
Genotype				
TT®		1.000		
TC	.443	1.419 (0.580–3.473)	.127	2.690 (0.754–9.591)
CC	.005*	4.400 (1.571–12.324)	.032*	4.417 (1.140–17.119)
Apri Score	.029*	4.084 (1.152–14.473)	.819	1.433 (0.065–31.387)
FIB‐4 index	.035*	1.674 (1.036–2.703)	.609	1.388 (0.396–4.867)

Abbreviations: C.I, Confidence interval; LL, Lower limit; OR, Odd's ratio; UL, Upper Limit.

^a^ All variables with *p* < .05 was included in the multivariate.

*Statistically significant at *p* ≤ .05.

**FIGURE 1:**
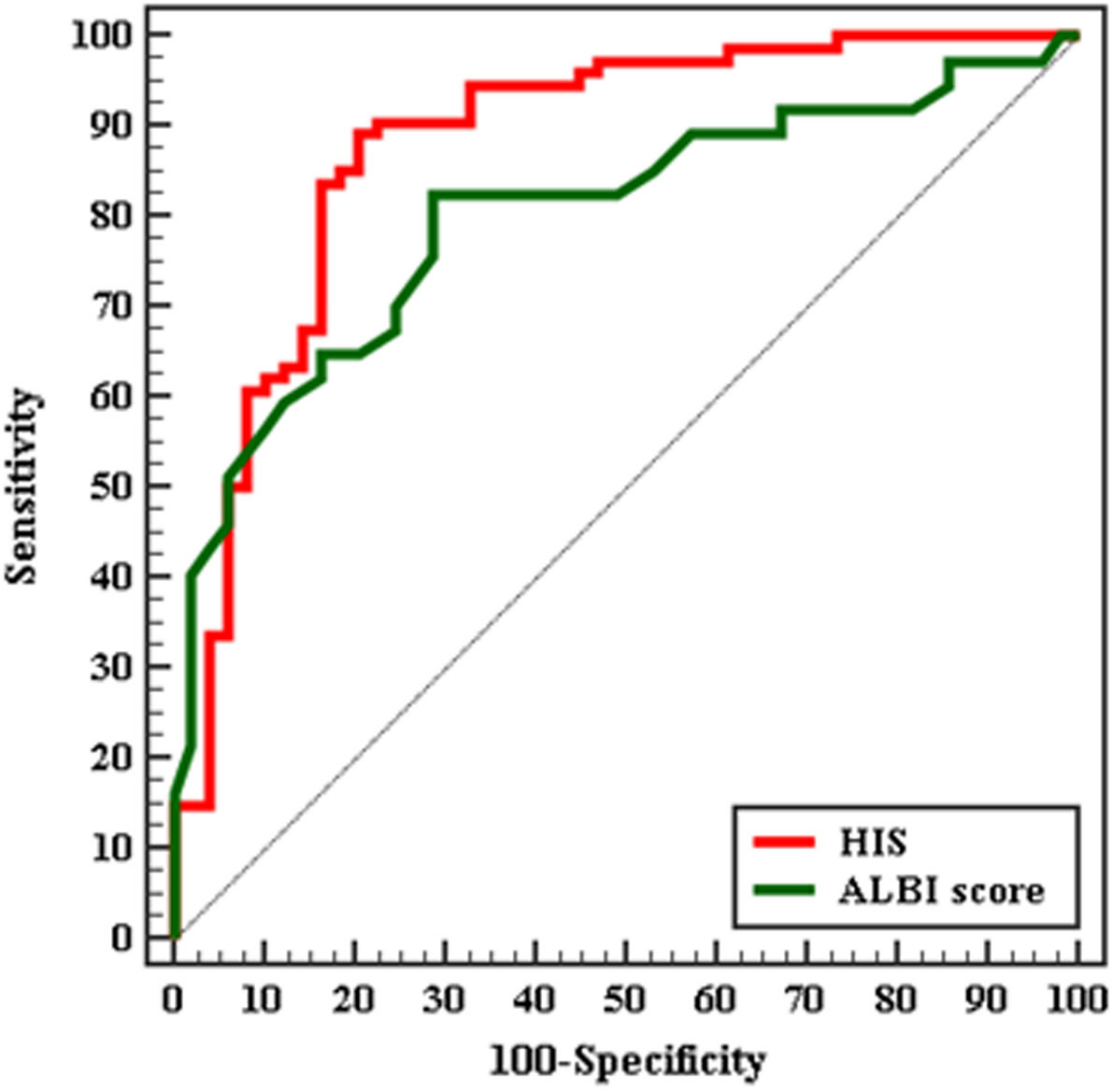
ROC curve for HSI and ALBI score to discriminate NAFLD early fibrosis (*n* = 74) from Control (*n* = 49)

HSI at cut off >38.82 was able to predict occurrence of fibrosis with sensitivity of 89.19%, and specificity 79.59% with AUC 0.881, while ALBI score at cut off > − 0.45 with sensitivity of 82.43%, and specificity 71.43% with AUC 0.802 (Figure 1).

**FIGURE 2:**
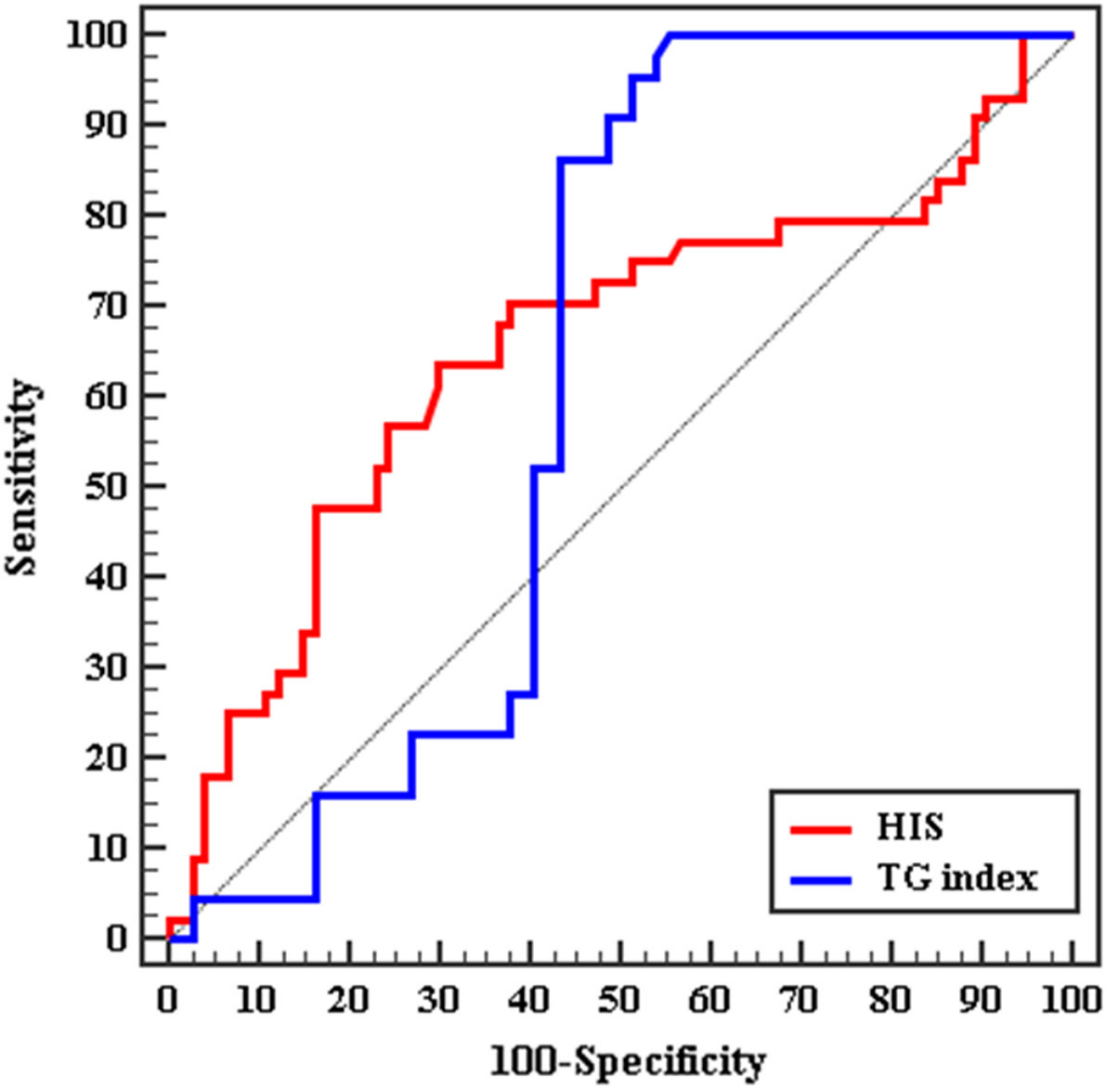
ROC curve for HIS and TG index to discriminate Significant fibrosis patients (*n* = 44) from NAFLD (early fibrosis) (*n* = 74)

HSI at cut off >44.14 was able to predict occurrence of advanced fibrosis at sensitivity of 70.45%, and specificity 62.16% with AUC 0.655, while TG index performed this at cut off >8.94 with sensitivity of 86.36%, and specificity 56.76% with AUC 0.626 (Figure 2).

## 8 DISCUSSION

In NAFLD‐related fibrosis, occurrence and progression is the most critical event in the natural history of this disease.^10^, ^11^ Recognition of the NAFLD cohort which is more susceptible for fibrosis progression is still an unmet need. ABCA1 genotypes were found to be strongly related to lipid metabolism and hepatic enzymology in NAFLD.^12^ Kolovou et al introduced a clear proof for correlation between ABCA1 genotypes, fatty liver and hepatic transaminases.^12^ Wang et al explained that wild homozygous and heterozygous models were lower in the NAFLD patients than in healthy individuals.^13^ It had been documented that *ABCA1* rs1800977 heterozygote was the most prevalent genotype in NAFLD patients.^13^

A linkage between ABCA1 polymorphisms and fibrosis progression in NAFLD patients was searched for in the current study. The results had showed a more prevalence of CC genotype of ABCA1 rs1800977 in late fibrosers (36.4%), than early fibrosers (13.5%), and control group (8.2%).

There was a significant difference in CC genotype distribution between NAFLD patients and late fibrosis (*p* = .005, OR = 4.400), and between late fibrosis and control (*p* = .002* OR = 7667) which indicates that CC genotype is more risky than TT by 4.4 times in NAFLD and 7.7 times in advanced fibrosis. There was a statistically significant difference in CC genotype distribution of ABCA1rs1800977 between NAFLD patients and early, late fibrosis and control groups in a recessive model CT + TT vs CC (*p* = .005 OR = 3.6 and 0.002 OR = 6.4), respectively.

C allele distribution showed statistically significant difference between early and late fibrosis, and between late fibrosis and control (*p* = .002 and .001), respectively. Results which might suggest a proof for a linkage between this gene polymorphism and danced fibrosis.

In the current study, advanced fibrosis in NAFLD patients was mainly reported in aged males (50.9 ± 9 years, 59.1%). A sensible finding as fibrosis progress needs more time to progress and premenopausal females are usually hormonal protected.^14^, ^15^

Obesity defined by BMI, weight loss and waist circumference had been suggested through lot of studies to be strong incriminator in fibrosis occurrence and progression^16^, ^17^, ^18^, ^19^; however, the current study had denied their role as determinants of advanced fibrosis. These results might be explained by the fact that obesity is responsible for increased neuroinflammatory process associating steatosis rather than frank liver fibrosis.^20^

In the current study DM, lipids, HSI, APRI, FIB4 scores and CC genotype of ABCA1 rs1800977 (*p* < .05) had been defined as the main factors affecting fibrosis progression in NAFLD patients. While multivariate Logistic regression analysis had nominated HSI, lipids, fibroscan and CC genotype of ABCA1 rs1800977 as independent predictors of fibrosis progression in NAFLD cases (*p* < .05).

In the same line, Fujii et al 2020, confirmed increase in cholesterol, low density lipoprotein, triglyceride and transaminases as a prognostic markers of non‐alcoholic steatohepatitis occurrence in NAFLD patients.^21^ Notably, triglycerides had been recently used as reliable markers also for extra‐hepatic sequela of NAFLD in aged patients.^22^

Tapper et al, clarified that APRI score more than one is the most significant predictor of advanced fibrosis in NAFLD cases.^23^ In the study performed by Hossain et al., diabetes mellitus and aminotransferases were said to be the only independent predictors of moderate‐to‐severe fibrosis.^24^ Also, diabetes mellitus and hypertension were the two mentioned parameters foreseeing occurrence of NAFLD‐related advanced fibrosis.^25^

Triglyceride glucose index (TG index) and NAFLD linkage is a well‐known causal relationship.^26^ Triglycerides accumulate in hepatic tissue when they exceeded the liver capacity to synthetize lipoproteins as in obese or insulin resistance. TG index was positively related to the severity of hepatic steatosis and the presence of liver fibrosis.^26^ In the current study, TG index was found to be significantly responsible for fibrosis occurrence and progression in NAFLD; however, applying multivariate logistic regression revoked this suggestion assuming that the main act of TG index is in fibrosis occurrence rather than progression.

In the current study, it was found that at cut off value >38.82 with area under the curve (AUC) 0.881 HIS had shown a sensitivity of 89.19% in prediction of fibrosis occurrence in NAFLD. While at cut off >44.14 was able to predict occurrence of advanced fibrosis at sensitivity of 70.45%, and specificity 62.16% with AUC 0.655.

This comes in accordance with Fedchuk et al. 2014, who used HSI in determining high grades of fibrosis with AUC of 0.65.^27^ Consistently, Eletreby et al, discussed HSI role in advanced fibrosis with a level of 45.35, sensitivity was 73.17%, specificity was 58.06%.^9^ Eletreby et al, documented that HSI can be used as a non‐invasive marker in screening of moderate steatohepatitis.^9^

Conclusively, this study declares that ABCA1 genotyping could be of great help in identifying cohorts with high risk of developing significant fibrosis with higher possibilities of progressing to cirrhosis with its morbid complications. Additionally, the linkage between dyslipidaemia and fibrosis progression had been remarkably delineated. Also, HSI would be a reliable diagnostic and prognostic marker of fibrosis in NAFLD patients. Accordingly, augmented models of both genetic, clinical and laboratory predictors of fibrosis should be more searched for more prediction accuracy.

More efforts are still needed to detect patients with higher liability of occurrence of advanced fibrosis. Defining this highly critical cohort takes the lead to more proper measures in their follow up for hindering progression of more fibrosis.

## AUTHOR CONTRIBUTIONS

**Samar Ghanem:** Formal analysis (equal); methodology (equal); writing – original draft (equal). **Maha Elsabaawy**: conceptalization (equal). **Mervat Abdelkareem:** Data curation (equal); validation (equal). **Marwa Helal:** Formal analysis (equal); investigation (equal); methodology (equal). **Warda Othman:** Data curation (equal); formal analysis (equal); resources (equal). **Mahitab Elsayed:** Resources (equal); validation (equal); visualization (equal). **Dalia Elsabaawy:** Formal analysis (equal); resources (equal); validation (equal). **Ashraf Elfert:** Formal analysis (equal); methodology (equal); project administration (equal); supervision (equal).

## FUNDING INFORMATION

The authors declare that no funds, grants or other support were received during the preparation of this manuscript.”

## CONFLICT OF INTEREST

The authors have no relevant financial or non‐financial interests to disclose.

## ETHICAL APPROVAL

This study was performed in line with the principles of the Declaration of Helsinki. Approval was granted by the Ethics Committee of National Liver Institute, Menoufia University no 20–0104.

## CONSENT TO PARTICIPATE AND PUBLISH

Written informed consent was obtained from all participants.

## Data Availability

All data is available online for all.
